# Comparison of the operative outcomes and learning curves between laparoscopic and “Micro Hand S” robot-assisted total mesorectal excision for rectal cancer: a retrospective study

**DOI:** 10.1186/s12876-021-01834-1

**Published:** 2021-06-07

**Authors:** Yanlei Wang, Guohui Wang, Zheng Li, Hao Ling, Bo Yi, Shaihong Zhu

**Affiliations:** Department of General Surgery, Third Xiangya Hospital, Central South University, 138 Tongzipo Street, Changsha, 410013 Hunan China

**Keywords:** Micro Hand S surgical robot system, Robotic surgery, Laparoscopic surgery, Total mesorectal excision, Rectal cancer

## Abstract

**Background:**

The Micro Hand S robot is a new surgical tool that has been applied to total mesorectal excision (TME) surgery for rectal cancer in our center. In this study, we compared the operative outcomes, functional outcomes and learning curves of the Micro Hand S robot-assisted TME (RTME) with laparoscopic TME (LTME).

**Methods:**

A total of 40 patients who underwent RTME and 65 who underwent LTME performed by a single surgeon between July 2015 and November 2018 were included in this retrospective study. Clinicopathologic characteristics, operative and functional outcomes, and learning curves were compared between the two groups. The learning curve was analyzed using the cumulative sum method and two stages (Phase 1, Phase 2) were identified and analyzed. All patients were followed up for at least 12 months.

**Results:**

The clinicopathologic characteristics of the two groups were similar. The learning curve was 17 cases for RTME and 34 cases for LTME. Compared with LTME, RTME was associated with less blood loss (148.2 vs. 195.0 ml, *p* = 0.022), and shorter length of hospital stay (9.5 vs. 12.2 days, *p* = 0.017), even during the learning period. With the accumulation of experience, the operative time decreased significantly from Phase 1 to Phase 2 (RTME, 360.6 vs. 323.5 min, *p* = 0.009; LTME, 338.1 vs. 301.9 min, *p* = 0.005), whereas other outcomes did not differ significantly.

**Conclusions:**

Micro Hand S robot-assisted TME is safe and feasible even during the learning period, with outcomes comparable to laparoscopic surgery but superior in terms of blood loss, length of hospital stay, and learning curve.

*Trial registration* Clinicaltrial.gov, NCT04836741, retrospectively registered on 5 April 2021.

**Supplementary Information:**

The online version contains supplementary material available at 10.1186/s12876-021-01834-1.

## Introduction

Since the laparoscope was first applied in colorectal diseases in 1991 [1], laparoscopic surgery for rectal cancer has become widely accepted. Laparoscopic surgery improves short-term outcomes (e.g., better cosmesis, faster recovery, and shorter hospital stay) compared to open surgery [2]. However, the laparoscope poses technical challenges for surgeons including poor flexibility, unavoidable physiologic tremor, and inferior ergonomics. The da Vinci surgical robot (Intuitive Surgical, Sunnyvale, CA, USA) overcomes the disadvantages of the laparoscope and is in clinical use; it shows excellent performance, especially in a narrow space. A recent meta-analysis found that robot-assisted rectal surgery was associated with lower rates of conversion and erectile dysfunction [3].

**Fig. 1:**
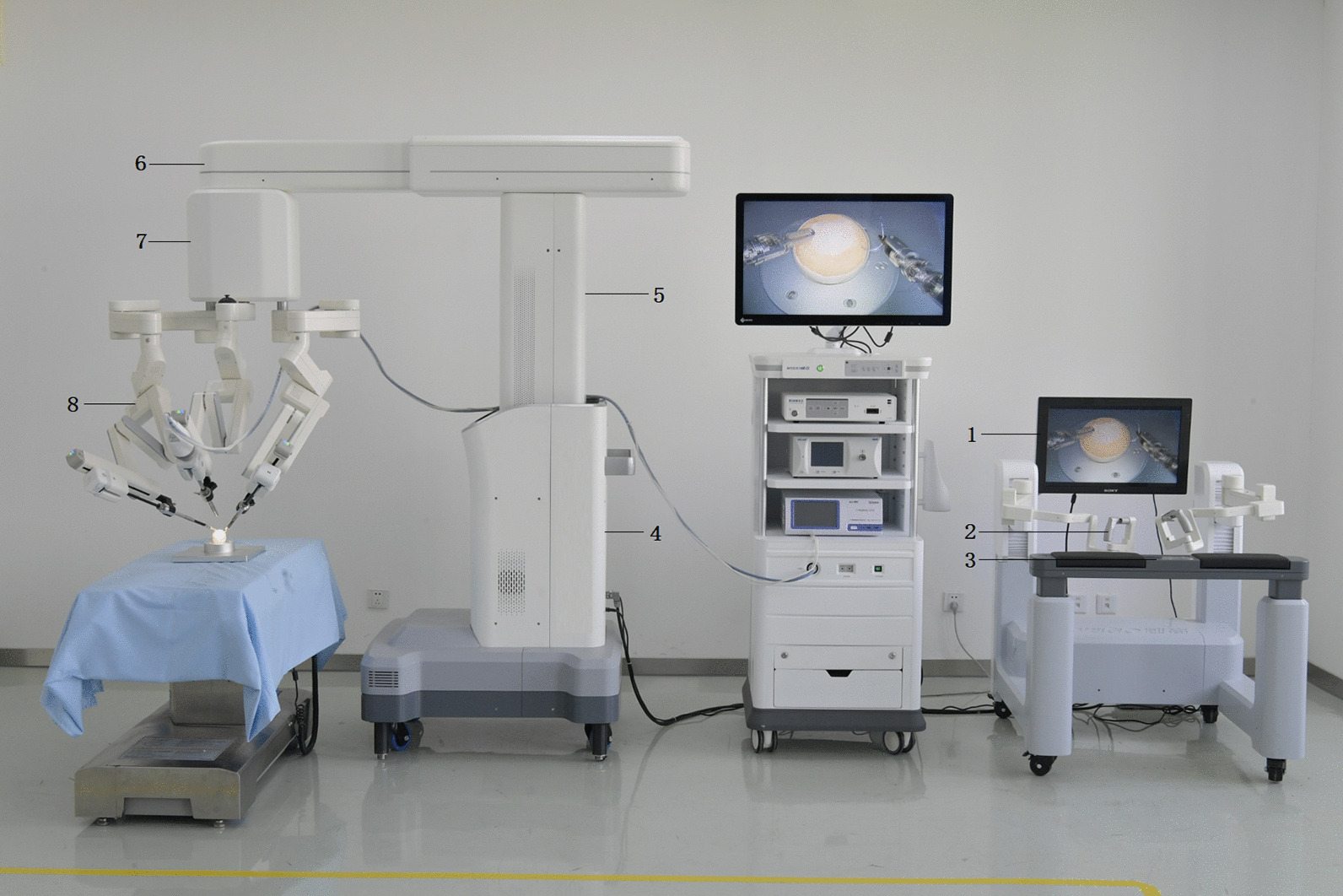
The Micro Hand S surgical robotic system. Surgeon console (right): (1) image display device, (2) master arm, (3) control panel. 3D imaging system (middle). Slave surgical cart (left): (4) electrical control system, (5) lifting column, (6) suspension passive arm, (7) swivel head, (8) slave arm

China is a country with a large population of rectal cancer patients; as such the prognosis for this disease is worse than in other countries with a lower incidence. To deliver better medical care to Chinese patients, our center in collaboration with Tianjin University developed the first Chinese surgical robot known as Micro Hand S (Wego, Qingdao, China) in 2013 (Fig. 1). This master–slave robot consists of a surgeon console, slave cart, and stereo image viewer [4]. Similar to the da Vinci surgical robot, the Micro Hand S robot is capable of 3-dimensional (3D) vision, motion scaling, tremor filtering, and wristed instrumentation with 7 degrees of freedom (DOF). Moreover, it has several innovative design elements including in miniaturization, an intuitive motion mapping strategy based on mechanical constraints, a decoupled design for multi-DOF devices, and a roll-pitch-roll form for the DOF arrangement of surgical instruments [4, 5]. The Micro Hand S has been used in clinical practice and its safety and feasibility have been preliminarily evaluated in cholecystectomy, gastric perforation repair, sleeve gastrectomy, and colectomy [4, 6].

Our center has performed total mesorectal excision (TME) using the Micro Hand S robot. In order to evaluate the efficacy and safety of Micro Hand S robot-assisted TME (RTME) surgery, for the first time, we compared the clinicopathologic characteristics and short-term outcomes (i.e., operative and functional outcomes and learning curves) of rectal cancer patients treated by RTME and laparoscopic TME (LTME).

## Materials and methods

### Patients and data

**Fig. 2:**
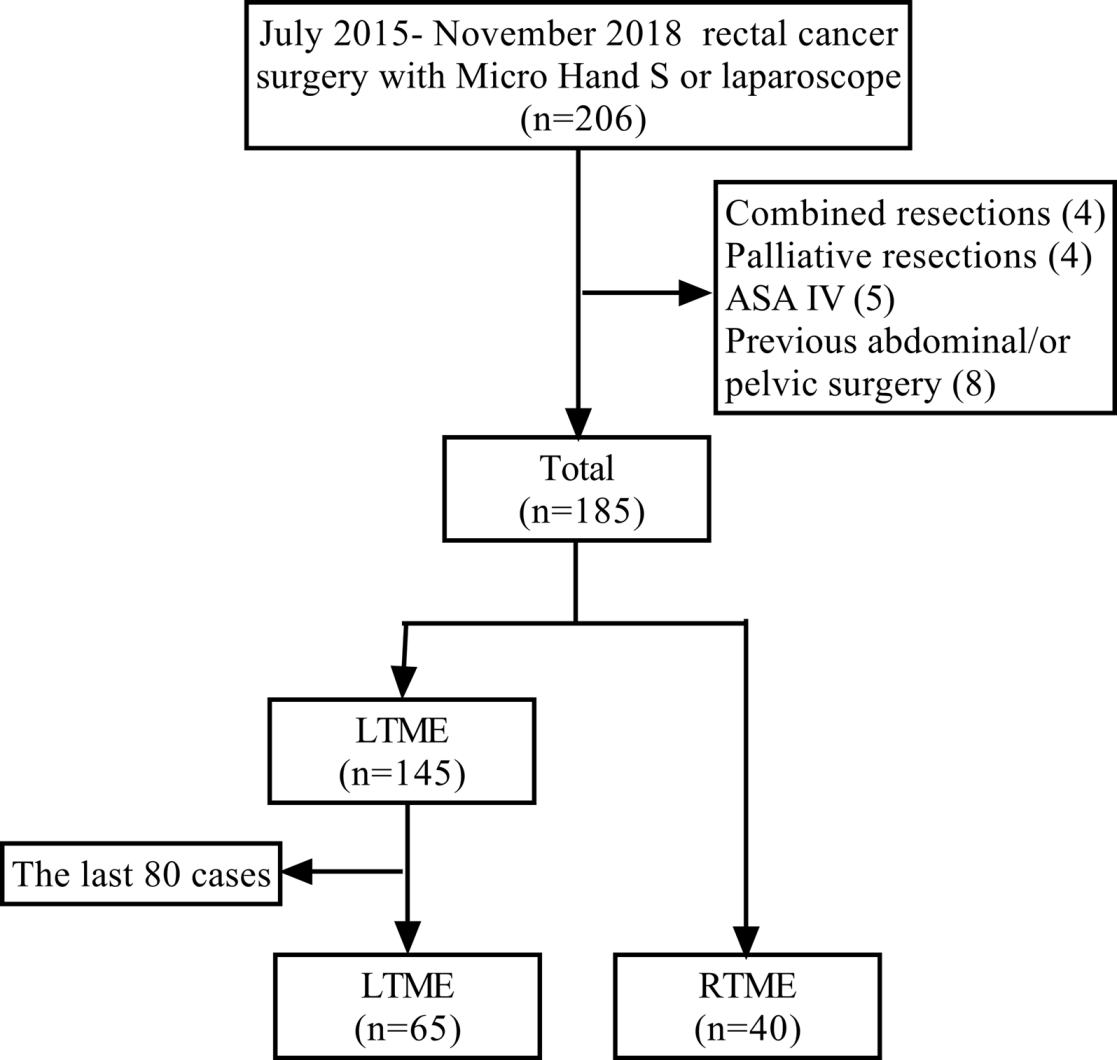
The flow chart of patient selection

We retrospectively analyzed data of patients with histologically confirmed rectal carcinoma. A total of 206 patients underwent TME surgery with the Micro Hand S robot or laparoscope at the Third Xiangya Hospital of Central South University, Changsha, China, between July 2015 and November 2018. We excluded patients with palliative or combined resections, distant metastasis, American Society of Anaesthesiologists (ASA) score ≤ 3, and previous history of abdominal/or pelvic surgery. The procedures were performed by a single surgeon. Prior to the first LTME surgery in July 2015, the surgeon had performed more than 300 open colorectal surgeries and assisted in more than 50 laparoscopic colorectal surgery. After performing more than 100 laparoscopic colorectal surgery and receiving training in the robotic surgical technique, the surgeon began conducting simple surgeries with the Micro Hand S robot including appendectomy, cholecystectomy, and gastric perforation repair, sleeve gastrectomy. The first RTME surgery using the Micro Hand S robot was performed in May 2017. In order to compare the learning curves with two surgical approaches, we included only consecutive cases: LTME (from July 2015 to January 2017) and RTME (from May 2017 to November 2018). Finally, 105 patients who underwent R/LTME for rectal cancer were included in this study (Fig. 2).

The patients had a standardized preoperative workup that included digital examination, rigid rectoscopy, complete colonoscopy, and thoracic-abdominal computed tomography (CT). Pelvic magnetic resonance imaging (MRI) was also performed to performed for clinical staging. All cases were discussed in multidisciplinary treatment meetings to decide on the administration of neoadjuvant chemoradiotherapy (CRT; 50.4 Gy in 25 fractions for 5 weeks with 5-fluorouracil chemotherapy). The indications for CRT were suspicious/positive circumferential resection margin (CRM) was and lymph nodes outside of the TME plane in the MRI [7]. The surgery was performed 6–8 weeks after completing CRT.

Data on patient characteristics along with operative, pathologic, and functional outcomes were collected. Conversion was defined as a change in treatment strategy to open surgery. The operative time (OT) was defined as the time from skin incision to skin closure. The status of resection margins was determined based on whether malignant cells were present ≤ 1 mm from the involved CRM or distal resection margin (DRM). The quality of the TME specimen was graded as complete, nearly complete, or incomplete [8]. Postoperative complications were graded according to the Clavien–Dindo classification, with grade III or higher considered as severe complication [9]. Functional outcomes including urinary and erectile functions were assessed in male patients using the International Prostate Symptom Score (IPSS) and the 5-item International Index of Erectile Function (IIEF-5) [10, 11] (Additional file 1). The patients filled out questionnaires at preoperatively, 3, 6, and 12 months postoperatively. All patients were followed up for at least 12 months. Local recurrence was defined as clinical, radiologic, or histopathologic evidence of any recurrent tumor in the anastomosis, pelvis, or perineum.

The study was approved by the Ethical Committee of the Third Xiangya Hospital and written informed consent was obtained from all patients. The study was reported adhering to STROBE guidelines.

### Surgical procedures

**Fig. 3:**
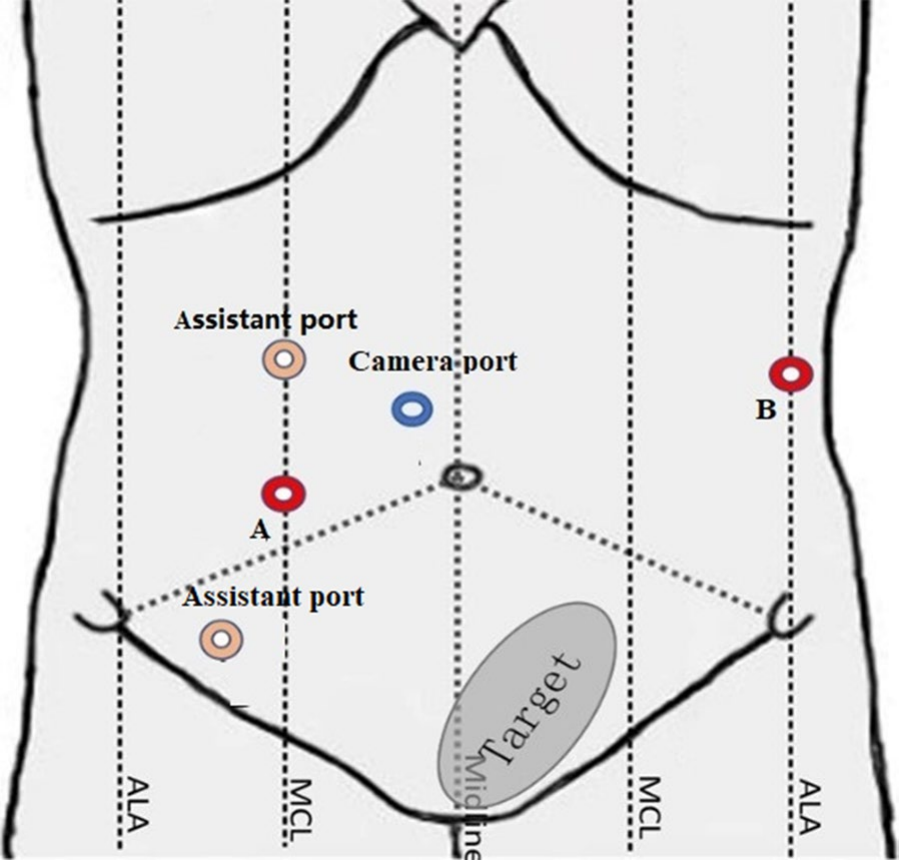
The arrangement of the surgical ports for RTME

We used a 3-armed Micro Hand S surgical robot. The arrangement of the ports is shown in Fig. 3. A 12-mm trocar was inserted through the supraumbilical port for the 3D camera (*camera port*). Two specialized 10-mm trocars were inserted through the two operating ports located 5–8 cm lateral to the right side of the umbilicus (*port A*) and 3–4 cm above the intersection of the umbilicus and left anterior axillary line (*port B*) to connect the right/left robotic instruments. Two assistant ports were created 10 cm below or above the right operating port.

A standardized robotic technique was adopted in all cases. After establishing of the pneumoperitoneum, the patients were placed in the lithotomy position with 20°-30° Trendelenburg and up to 15°–20° right tilt. The Micro Hand S surgical robot was docked on the left side of the patient as previously described [4]. Arm 1 was equipped with ultrasonic shears, or mono-polar scissors and arm 2 was equipped with the grasper. First, we ligated the inferior mesenteric vessels and then mobilized the left colon and sigmoid colon using a medial-to-lateral approach. The splenic flexure was taken down as needed. Second, we dissected the pelvic cavity, firstly the posterior dissection, then the lateral, and lastly the anterior dissection. The hypogastric nerves were identified and carefully preserved. Once the dissection was completed, the robot was undocked and the rectum was transected using a cutting stapler under laparoscopic guidance. The specimen was extracted through a 3–4 cm abdominal incision. A circular stapler was inserted through the anus and an intracorporeal end-to-end anastomosis was created laparoscopically. Additional details are shown in Additional file 2.

Laparoscopic surgery was performed following the same steps.

### Statistical analysis

Data were analyzed using SPSS v20.0 software (SPSS Inc, Chicago, IL, USA). Continuous variables are expressed as mean ± standard deviation and comparisons between groups were performed with the Student’s t test. Categorical variables are expressed as frequencies (percentages) and comparisons between groups were performed with Pearson’s chi-squared test or Fisher’s exact test. Differences were considered statistically significant at *p* < 0.05.

The cumulative sum (CUSUM) method is a powerful tool for early detection of trends in data [12]. We used this method to analyze the learning curve of the OT. Cases treated by each approach were arranged in chronological order. For each approach, the CUSUM_OT_ for the first case was the difference between the OT for the first case and mean OT of all cases. The CUSUM_OT_ of the second case was the CUSUM_OT_ of the previous case added to the difference between the OT of the second case and mean OT. The procedure was repeated for each case except for the last one, which was calculated as zero. The learning curve was considered complete when a peak point was observed in the CUSUM plot.

## Results

### Patient characteristics and clinicopathological outcomes

There were 40 patients in the RTME group and 65 in the LTME group. All patients were successfully discharged. There were no significant differences in age, sex, body mass index (BMI), and ASA score between the two groups (Table 1). The tumor location tended to be higher in the RTME group than in the LTME group (8.2 vs 7.5 cm, *p* = 0.299). Low anterior resection (LAR) was the main procedure performed in both groups (95.0 vs 93.8%, *p* = 1.000). The OT was slightly longer with RTME than with LTME, but the difference was not significant (339.3 vs 320.8 cm, *p* = 0.070). The RTME group had less blood loss (140.9 vs 188.0 ml, *p* = 0.001) and a shorter length of hospital stay (9.2 vs 11.9 days, *p* = 0.001) than the LTME group. There was one conversion in the RTME group (2.5%) due to high BMI and 3 in the LTME group (4.6%) because of low tumor location or large tumors. None of the patients in either group had DRM involvement, while two in the RTME group were considered to have CRM involvement (5.0 vs 0%, *p* = 0.143). The quality of TME specimens was comparable in the two groups (complete/nearly complete/incomplete, 77.5/15.0/7.5% for the RTME group and 73.8/18.5/7.7% for the RTME group, *p* = 0.897).

**Table 1:** Patient characteristics and clinicopathological outcomes between RTME and LTME

	RTME (n = 40)	LTME (n = 65)	*p* value
Age (year)	57.4 ± 9.5	60.2 ± 10.8	0.178
Male	20 (50.0)	31 (47.7)	0.818
BMI (kg/m^2^)	21.8 ± 2.9	22.2 ± 4.2	0.577
ASA (I/II/III)	19/16/5 (47.5/40.0/12.5)	23/28/14 (35.4/43.1/21.5)	0.353
Distance from AV (cm)	8.2 ± 2.9	7.5 ± 3.2	0.299
CRT	5 (12.5)	5 (7.7)	0.415
*Procedure type*			1.000
LAR	38 (95.0)	61 (93.8)	
APR	2 (5.0)	4 (6.2)	
Operative time (min)	339.3 ± 45.3	320.8 ± 52.8	0.070
Blood loss (ml)	140.9 ± 68.5	188.0 ± 73.0	0.001
Conversion	1 (2.5)	3 (4.6)	1.000
Protective ileostomy	22 (55.0)	31 (51.7)	0.744
Hospital stay (day)	9.2 ± 3.8	11.9 ± 3.9	0.001
Tumor size (cm)	3.4 ± 1.4	3.8 ± 1.5	0.240
Retrieved lymph node	15.3 ± 4.9	15.6 ± 5.3	0.774
pTNM (I/II/III)	4/11/25 (10.0/27.5/62.5)	13/15/37 (20.0/23.1/56.9)	0.395
DRM (cm)	2.4 ± 1.3	2.5 ± 1.5	0.804
Involved DRM	0	0	–
Involved CRM	2 (5.0)	0	0.143
*Grade of differentiation*			0.846
Poor	12 (30.0)	15 (23.1)	
Moderate	17 (42.5)	30 (46.2)	
Well	8 (20.0)	13 (20.0)	
Others	5 (12.5)	7 (10.7)	
*Quality of TME*			0.897
Complete	31 (77.5)	48 (73.8)	
Nearly complete	6 (15.0)	12 (18.5)	
Incomplete	3 (7.5)	5 (7.7)	

*BMI* body mass index, *ASA* American Society Anesthesia, *AV* anal verge, *CRT* chemoradiation therapy, *LAR* low anterior resection, *APR* abdominal perineal resection, *DRM* distal resection margin, *CRM* circumferential resection margin, *TME* total mesorectal excision. Values are presented as mean ± standard deviation, n or n (%)

The follow-up period was 12–17 months. During follow-up, two local recurrences were observed in the LTME group.

### Postoperative complications

In total, 35 complications were reported including 12 (30.0%) in the RTME group and 23 (35.4%) in the LTME group (Table 2). The most common complications were wound complications (6.7%). According to the Clavien–Dindo classification, no significant difference was observed in the two groups (*p* = 0.742). In the RTME group, there were 3 severe complications (≥ grade III): one case of anastomotic leakage (grade IIIa) and two cases of anastomotic hemorrhage (grade IIIb) for which endoscopic treatment was carried out. In the LTME group, there was one case of anastomotic leakage (grade IIIb) and two cases of anastomotic hemorrhage (grade IIIa) that were similarly treated. One patient in the LTME group who experienced pulmonary failure (grade IVa) was admitted to the intensive care unit.

**Table 2:** Postoperative complications between RTME and LTME

	RTME (n = 40)	LTME (n = 65)	*p* value
*Overall complication*	12 (30.0)	23 (35.4)	0.570
Wound complication	2 (5.0)	5 (7.7)	0.706
Fluid collection	3 (7.5)	3 (4.6)	0.672
Ileus	1 (2.5)	4 (6.2)	0.647
Urinary retention	2 (1.9)	3 (4.6)	1.000
Pneumonia	0	2 (3.1)	0.524
Pulmonary failure	0	1 (1.5)	1.000
Anastomotic bleeding	2 (1.9)	3 (4.6)	1.000
Anastomotic leakage	1 (2.5)	1 (1.5)	1.000
Intraabdominal abscess	0	1 (1.5)	1.000
Anterior resection syndrome	1 (2.5)	0	0.381
C–D grade (III–V)	3 (7.5)	4 (6.2)	1.000

*C–D* Clavien–Dindo. Values are presented as n (%)

### Functional outcomes

**Fig. 4:**
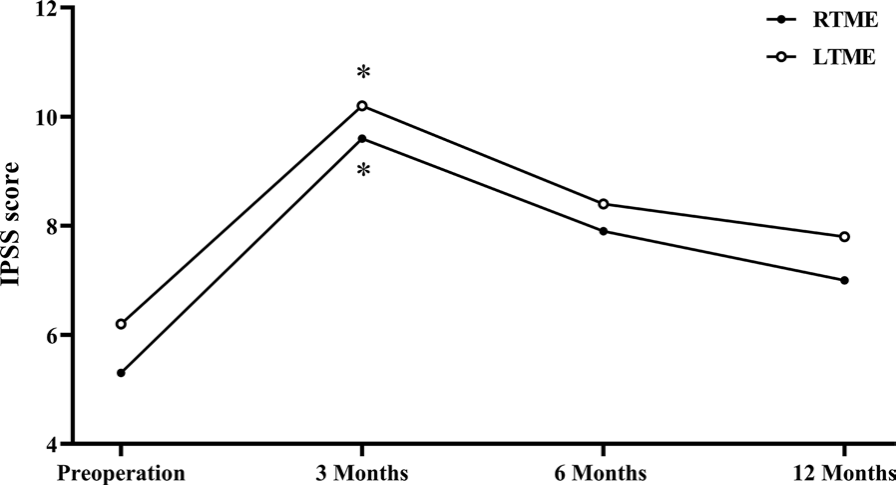
The IPSS score for RTME and LTME in male patients. *p < 0.05 for difference in mean scores between preoperatively and each time point

**Fig. 5:**
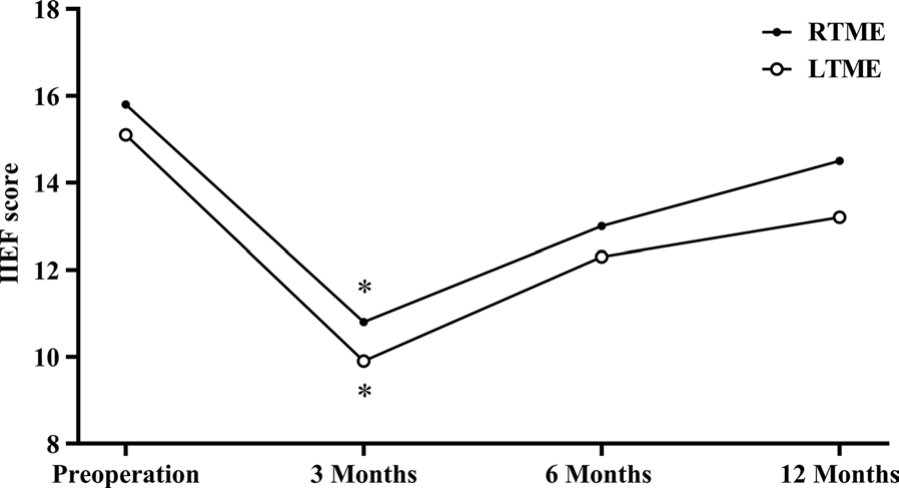
The IIEF-5 score for RTME and LTME in male patients. *p < 0.05 for difference in mean scores between preoperatively and each time point

In total, 35/51 male patients (68.6%) responded to the questionnaires on urinary and erectile functions (Table 3). The IPSS score increased postoperatively and gradually decreased in the two groups, with no significant intergroup differences at any time point (Table 3; Fig. 4). The IPSS score decreased to the preoperative level at 6 months postoperatively in the two groups (5.3 vs. 7.9, *p* = 0.091; 6.2 vs. 8.4,* p* = 0.072). The IIEF-5 score also decreased to the preoperative level at 6 months postoperatively in both groups with no intergroup difference was observed (Table 3; Fig. 5).

**Table 3:** Comparison of functional outcomes between RTME and LTME in male patients

	RTME (n = 13)	LTME (n = 22)	*p* value
*IPSS score*			
Pre-operation	5.3 ± 3.5	6.2 ± 3.6	0.494
3 Months	9.6 ± 5.0	10.2 ± 5.3	0.759
*p* (baseline vs.)	0.019	0.006	
6 months	7.9 ± 4.0	8.4 ± 4.3	0.743
*p* (baseline vs.)	0.091	0.072	
12 months	7.0 ± 3.5	7.8 ± 3.9	0.557
*p* (baseline vs.)	0.230	0.168	
*IIEF-5 score*			
Pre-operation	15.8 ± 5.6	15.1 ± 5.3	0.740
3 Month	10.8 ± 4.0	9.9 ± 5.1	0.609
*p* (baseline vs.)	0.015	0.002	
6 months	13.0 ± 4.4	12.3 ± 5.3	0.679
*p* (baseline vs.)	0.174	0.080	
12 months	14.5 ± 5.4	13.2 ± 5.2	0.483
*p* (baseline vs.)	0.575	0.235	

*IPSS* International Prostate Symptom Score, *IIEF* International Index of Erectile Function. Values are presented as mean ± standard deviation

### Learning curves

#### Determining the learning curve

**Fig. 6:**
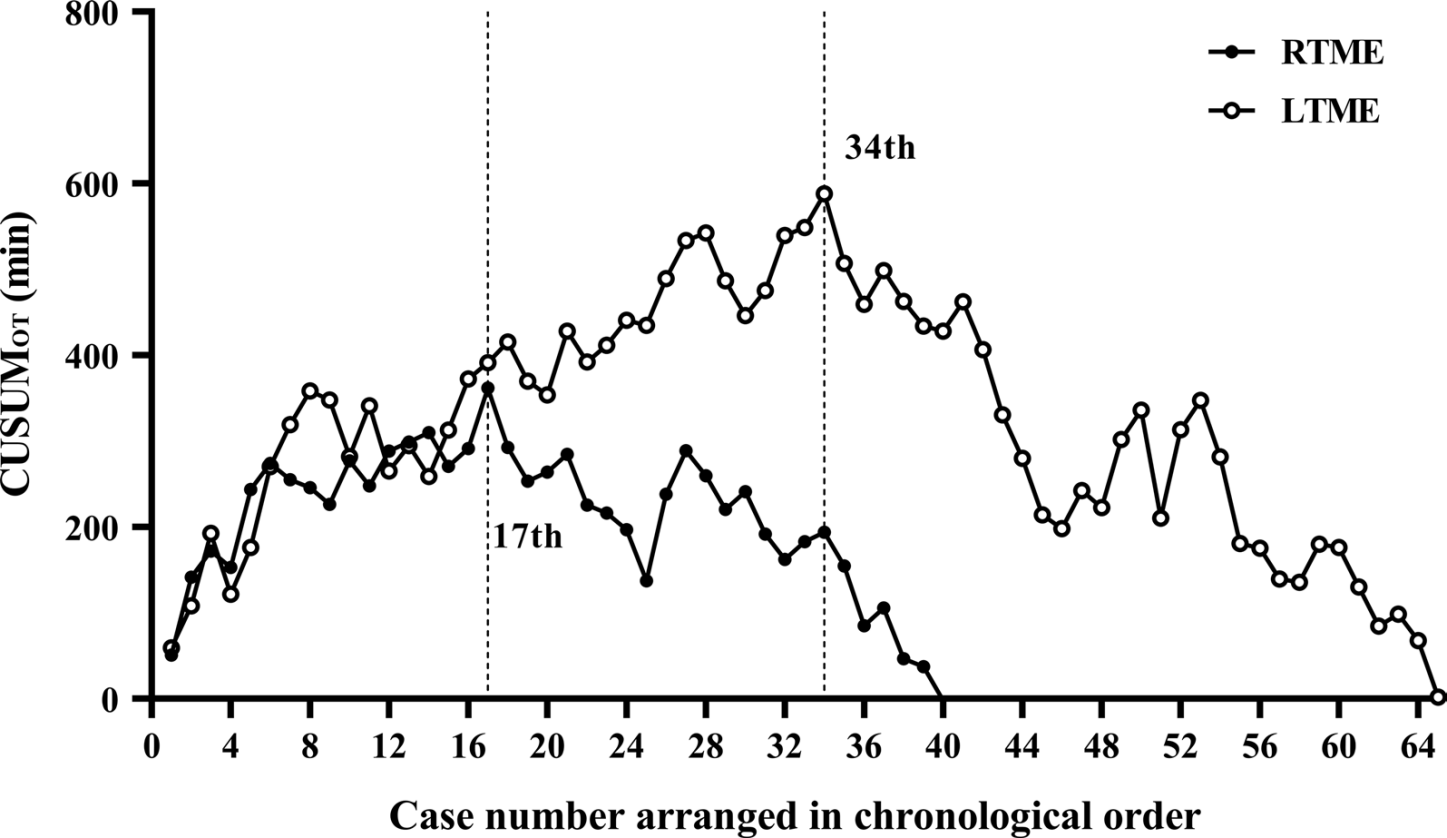
The learning curves for RTME and LTME. The curve of RTME reached the peak point at the 17th case; and the curve of LTME reached the peak point at the 34th case

According to the CUSUM analysis, OT increased in the initial phase, reaching a peak before gradually decreasing (Fig. 6). The learning curve was therefore divided into Phase 1 (initial learning period, 1st–17th case) versus Phase 2 (the post learning period, 18th–40th case) for the RTME group, and Phase 1 (the initial learning period, 1st–34th case) versus Phase 2 (the post learning period, 35th–65th case) for the LTME group.

#### Learning curve phases: RTME versus LTME

The two groups showed similar characteristics in Phase 1 (Table 4). The blood loss was significantly lower in the RTME group than that in the LTME group (148.2 vs 195.0 ml, *p* = 0.022). Similarly, the length of hospital stay was shorter with RTME than with LTME (9.5 vs 12.2 days, *p* = 0.017). As for the rates of severe complications (grade III–V), the two groups were similar (5.9 vs. 5.9%, *p* = 1.000). The quality of TME specimens was comparable between the two groups (complete/nearly complete/incomplete: 70.6/23.5/5.9% for the RTME group and 67.6/23.5/8.8% for the LTME group, *p* = 0.933). In Phase 2, all of the parameters showed similar results to Phase 1 (Table 4).

**Table 4:** Comparison of the learning curve phases: RTME versus LTME

	Phase 1 (n = 51)			Phase 2 (n = 54)		
	RTME (n = 17)	LTME (n = 34)	*p* value	RTME (n = 23)	LTME (n = 31)	*p* value
Age (year)	56.2 ± 9.5	60.1 ± 10.1	0.189	58.3 ± 9.6	60.3 ± 11.7	0.500
Male	8 (47.1)	16 (47.1)	1.000	12 (52.2)	15 (48.4)	0.783
BMI (kg/m^2^)	21.8 ± 2.9	22.8 ± 4.7	0.414	21.8 ± 3.0	21.5 ± 3.5	0.735
ASA (I/II/III)	6/9/2 (35.3/52.9/11.8)	13/15/6 (38.2/44.1/17.7)	0.793	13/7/3 (56.5/30.4/13.1)	10/13/8 (32.3/41.9/25.8)	0.187
Distance from AV (cm)	8.1 ± 3.1	7.6 ± 3.2	0.588	8.3 ± 2.9	7.5 ± 3.4	0.375
CRT	1 (5.9)	3 (8.8)	1.000	4 (17.4))	2 (6.5)	0.384
*Procedure type*			1.000			1.000
LAR	16 (94.1)	32 (94.1)		22 (95.7)	29 (93.5)	
APR	1 (5.9)	2 (5.9)		1 (4.3)	2 (6.5)	
Operative time (min)	360.6 ± 40.8	338.1 ± 47.2	0.100	323.5 ± 42.6	301.9 ± 52.8	0.114
Blood loss (ml)	148.2 ± 73.8	195.0 ± 62.4	0.022	135.4 ± 65.4	180.3 ± 83.5	0.037
Conversion	1 (5.9)	2 (5.9)	1.000	0	1 (3.2)	1.000
Protective ileostomy	11 (64.7)	19 (63.3)	0.925	11 (47.8)	12 (40.0)	0.569
Hospital stay (day)	9.5 ± 3.7	12.2 ± 3.5	0.017	9.0 ± 4.0	11.6 ± 4.4	0.029
C–D grade (III–V)	1 (5.9)	2 (5.9)	1.000	2 (8.7)	2 (6.5)	1.000
Tumor size (cm)	3.3 ± 1.2	3.5 ± 1.4	0.602	3.5 ± 1.6	4.0 ± 1.6	0.221
Retrieved lymph node	15.2 ± 4.8	16.7 ± 5.7	0.369	15.4 ± 5.0	14.5 ± 4.8	0.485
pTNM (I/II/III)	1/4/12 (5.9/23.5/70.6)	5/7/22 (14.7/20.6/64.7)	0.652	3/7/13 (13.1/30.4/56.5)	8/8/15 (25.8/25.8/48.4)	0.515
DRM (cm)	2.4 ± 1.3	2.7 ± 1.5	0.545	2.4 ± 1.4	2.3 ± 1.5	0.757
Involved DRM	0	0	–	0	0	–
Involved CRM	1 (5.9)	0	0.333	1 (4.3)	0	0.426
*Quality of TME*			0.933			0.858
Complete	12 (70.6)	23 (67.6)		19 (82.6)	25 (80.6)	
Nearly complete	4 (23.5)	8 (23.5)		2 (8.7)	4 (12.9)	
Incomplete	1 (5.9)	3 (8.8)		2 (8.7)	2 (6.5)	

*BMI* body mass index, *ASA* American Society Anesthesia, *AV* anal verge, *CRT* chemoradiation therapy, *LAR* low anterior resection, *APR* abdominal perineal resection, *DRM* distal resection margin, *CRM* circumferential resection margin, *TME* total mesorectal excision. Values are presented as mean ± standard deviation or n (%)

#### Learning curve phases: Phase 1 versus Phase 2

In the RTME group, with the accumulation of experience, the cases in Phase 2 had a shorter OT than those in Phase 1 (323.5 vs 360.6 min, *p* = 0.009) (Table 5). Although blood loss showed a decreasing trend from Phase 1 to Phase 2, it did not reach statistical difference (148.2 vs. 135.4 ml, *p* = 0.566). The length of hospital stay did not differ between the two phases (9.5 vs 9.0 days, *p* = 0.644). The rates of severe complications were comparable (5.9 vs 8.7%, *p* = 1.000). The quality of TME was similar between the two phases (*p* = 0.423), but the rates of complete TME tended to increase (70.6 vs 82.6%), whereas the opposite trend was observed for the rate of nearly complete TME (23.5 vs 8.7%). In the LTME group, all of the characteristics showed similar results to the RTME group (Table 5).

**Table 5:** Comparison of the learning curve phases: Phase 1 versus Phase 2

	RTME (n = 40)			LTME (n = 65)		
	Phase 1 (n = 17)	Phase 2 (n = 23)	*p* value	Phase 1 (n = 34)	Phase 2 (n = 31)	*p* value
Age (year)	56.2 ± 9.5	58.3 ± 9.6	0.500	60.9 ± 10.0	60.3 ± 11.7	0.941
Male	8 (47.1)	12 (52.2)	0.749	16 (47.1)	15 (48.4)	0.915
BMI (kg/m^2^)	21.8 ± 2.9	21.8 ± 3.0	0.962	22.8 ± 4.7	21.5 ± 3.5	0.217
ASA (I/II/III)	6/9/2 (35.3/52.9/11.8)	13/7/3 (56.5/30.4/13.1)	0.337	13/15/6 (38.2/44.1/17.7)	10/13/8 (32.3/41.9/25.8)	0.711
Distance from AV (cm)	8.1 ± 3.1	8.3 ± 2.9	0.856	7.6 ± 3.2	7.5 ± 3.4	0.897
CRT	1 (5.9)	4 (23.0)	0.373	3 (8.8)	2 (6.5)	1.000
*Procedure type*			1.000			1.000
LAR	16 (94.1)	22 (95.7)		32 (94.1)	29 (93.5)	
APR	1 (5.9)	1 (4.3)		2 (5.9)	2 (6.5)	
Operative time (min)	360.6 ± 40.8	323.5 ± 42.6	0.009	338.1 ± 47.2	301.9 ± 52.8	0.005
Blood loss (ml)	148.2 ± 73.8	135.4 ± 65.4	0.566	195.0 ± 62.4	180.3 ± 83.5	0.422
Conversion	1 (5.9)	0	0.425	2 (5.9)	1 (3.2)	1.000
Protective ileostomy	11 (64.7)	11 (47.8)	0.289	19 (63.3)	12 (40.0)	0.071
Hospital stay (day)	9.5 ± 3.7	9.0 ± 4.0	0.644	12.2 ± 3.5	11.6 ± 4.3	0.544
C–D grade (III–V)	1 (5.9)	2 (8.7)	1.000	2 (5.9)	2 (6.5)	1.000
Tumor size (cm)	3.3 ± 1.2	3.5 ± 1.6	0.707	3.5 ± 1.4	4.0 ± 1.6	0.166
Retrieved lymph node	15.2 ± 4.8	15.4 ± 5.0	0.892	16.7 ± 5.7	14.5 ± 4.8	0.099
pTNM (I/II/III)	1/4/12 (5.9/23.5/70.6)	3/7/13 (13.1/30.4/56.5)	0.613	5/7/22 (14.7/20.6/64.7)	8/8/15 (25.8/25.8/48.4)	0.377
DRM (cm)	2.4 ± 1.3	2.4 ± 1.4	0.964	2.7 ± 1.5	2.3 ± 1.5	0.346
Involved DRM	0	0	–	0	0	–
Involved CRM	1 (5.9)	1 (4.3)	1.000	0	0	–
*Quality of TME*			0.423			0.477
Complete	12 (70.6)	19 (82.6)		23 (67.6)	25 (80.6)	
Nearly complete	4 (23.5)	2 (8.7)		8 (23.5)	4 (12.9)	
Incomplete	1 (5.9)	2 (8.7)		3 (8.8)	2 (6.5)	

*BMI* body mass index, *ASA* American Society Anesthesia, *AV* anal verge, *CRT* chemoradiation therapy, *LAR* low anterior resection, *APR* abdominal perineal resection, *DRM* distal resection margin, *CRM* circumferential resection margin, *TME* total mesorectal excision. Values are presented as mean ± standard deviation or n (%)

## Discussion

In this study, the operative and functional outcomes and learning curves for RTME versus LTME were compared in the initial stage. The results showed that robotic surgery was associated with less blood loss, shorter length of hospital stay, and an accelerated learning curve compared to laparoscopic surgery without other outcomes being compromised.

Only a small number of patients received neoadjuvant CRT in this study because selective neoadjuvant CRT was administered. For patients with tumors and/or lymph nodes located in the TME plane without suspected or confirmed CRM involvement, standard TME surgery can be curative and neoadjuvant CRT is not essential [13]. Moreover, neoadjuvant CRT can induce tissue edema, which increases the difficulty of pelvic dissection and the risk of intra- and postoperative complications [14].

The cases included in this study were still in the initial stage. Patients with favorable clinical profiles tended to be selected when applying a new technique with which the surgeon has minimal experience. However, this was not considered in the current study. The patients’ characteristics were similar between Phase 1 and Phase 2, and the distance of the tumor from anal verge—which dictates the depth of dissection in the pelvis and surgical difficulty [15]—did not differ between the phases.

As OT is usually taken as a surrogate for the learning curve, in this study the CUSUM method for OT was used to determine the learning curve. We observed an accelerated learning curve for robotic surgery, with the surgeon achieving proficiency after just 17 cases. Similarly, previous studies have found that the learning curve for rectal cancer surgery with the da Vinci robot was 15–50 cases [16–18]. The accelerated learning curve for RTME can be explained as follows. (1) The surgeon’s previous experience with laparoscopic surgery may have facilitated the learning curve of robotic surgery because the two procedures followed the same principles and were performed in a similar surgical environment [19]. (2) Similar to the da Vinci robot, the surgeon console of the Micro Hand S robot was user-friendly manner in that it imitated the motion of the surgeon's hand (Additional file 2: Video S1), allowing quick mastery of its operation. (3) Unlike the long and rigid laparoscope, the Micro Hand S robot is equipped with an endo-wrist instrument with 7 DOF, 90° articulation, and 540° rotation; this feature facilitates instrument manipulation, especially in the narrow and deep pelvic cavity, which is difficult to navigate with a laparoscope.

The robotic surgery had less intraoperative blood loss than the laparoscopic surgery (140.9 vs 188.0 ml); this difference remained significant during the initial learning period (Phase 1) (148.2 vs 195.0 ml). However, the learning curve had no significant effect on blood loss (Table 5). In our experience, with a high-resolution view, the surgeon can easily identify the avascular plane and perform the dissection more smoothly. Moreover, the Micro Hand S robot is equipped with a motion scaling solution [20] that allows the surgeon to downscale the instruments’ motion at 1:3, 1:6, and 1:10 ratios as needed. These features allow the accurate manipulations and thus reduce blood loss as well as the risk of bowel injury, leading to a more rapid recovery and shorter hospital stay.

The incidence of perioperative complications—especially severe complication—is an important indicator of the safety of the surgical procedure. The rate of severe complications in robotic surgery was 7.5%, similar to 7.0% of other meta-analysis [21]. Two anastomotic leakages were treated without protective ileostomy, which can effectively divert the feces and may reduce anastomotic leakage rate [22] although this point is controversial. During the learning period, safety was always a concern. We showed that the rate of severe complications in robotic surgery was comparable between Phase 1 and Phase 2, confirming the safety of this method during the learning period.

The grade of mesorectal excision is a crucial indicator of the quality of rectal resection and can predict local recurrence. An intact mesorectum is always considered ideal and in the current study the rate was 77.5% for robotic surgery. This is similar to the rate of 77.8% reported in a meta-analysis including four randomized controlled trials [3]. Furthermore, we found that the learning curve had a significant impact on this parameter: for the robotic surgery, the rate was 70.6% in Phase 1 and rapidly increased to 82.6% in Phase 2. Incomplete TME is associated with a higher risk of local recurrence, but no significant difference was observed between patients with nearly complete and complete TME [23, 24]. A two grade system was therefore proposed: adequate TME (corresponding to complete and nearly complete excision) and inadequate TME (incomplete excision). The rate of adequate TME was 92.5% in robotic surgery and 92.3% in laparoscopic surgery. Consistent with our results, two randomized controlled trials reported rates of 89.4% (211/236) and 98.5% (65/66) [25, 26]. Additionally, we found that the learning curve had no impact on the rate of adequate TME, which was 94.1% in Phase 1 and 91.3% in Phase 2 for robotic surgery. This suggests that during the learning period, the quality of TME was not compromised, which could be attributed to the surgeon’s previous experience in laparoscopic surgery. During the learning period, such experience can transmit into rapid mastery of robotic surgery, especially in the identification of anatomic structures and dissection of the avascular plane between the presacral fascia and fascia propria of the rectum without injury to the mesorectum in the narrow pelvic cavity, which ensures an adequate excision.

A CRM with a distance of > 1 mm is the accepted standard for gauging the quality of the surgery. In the current study, there were two cases with CRM involvement (< 1 mm) (5.0%) in robotic surgery, which is comparable to the rates reported in previous meta-analyses (2.9–6.6%) [27, 28]. Both patients were male with mid-low and advanced tumors (T4 and N1 stages). All these characteristics are risk factors for involved CRM [29]. Both patients received postoperative CRT and no local recurrence was observed during the follow-up. However, local recurrence occurred in two patients with a clear CRM or DRM who had complete TME. In both cases the lymph nodes were involved and < 12 lymph nodes were dissected, suggesting that the local recurrence was due to the inadequate number of harvested lymph nodes.

Functional outcomes were assessed based on urinary and erectile function functions. Because of insufficient data on female patients, the analysis included only male patients. Functional scores declined after operation but progressively improved, with no significant difference between the two groups. The scores at 6 months postoperatively reached the preoperative level in both groups. As shown by Luca et al. [30], although the features of the surgical robot were helpful for identifying and preserving pelvic autonomic nerves, robotic surgery showed no superiority over laparoscopic surgery for performing nerve-sparing rectal cancer surgery.

The limitations of this study were as follows. Firstly, the study had a small sample size; the efficacy and safety of robotic surgery must be validated in a larger cohort. Secondly, as a retrospective study the selection bias did exist; for example, given the learning period we did not include complicated cases such as ultralow rectal cancers, which likely undermined the objectivity of the results. The advantages of robotic surgery will be fully established by including such cases. Third, the accelerated learning curve was for a surgeon with extensive experience in laparoscopic surgery, and may not apply to surgeons without laparoscopic experience. In addition, long-term oncologic outcomes were not included and should be evaluated in the future.

## Conclusion

The present study indicates that TME assisted by the Micro Hand S robot is safe and feasible for rectal cancer, even during the learning period. The Micro Hand S robot may be an alternative for rectal cancer. A randomized controlled trial needs to be conducted in the future to further confirm our results. The long-term oncologic parameters will also be assessed in future studies.

## Supplementary Information

**Additional file 1.** The questionnaires on functional outcomes.

**Additional file 2.** The details during the operation.

## Data Availability

The datasets generated and/or analyzed during the current study are available from the corresponding author on reasonable request.

## Abbreviations

ASA: American Society of Anesthesiology
APR: Abdominal perineal resection
AV: Anal verge
BMI: Body mass index
CRM: Circumferential resection margin
CRT: Chemoradiation therapy
CUSUM: Cumulative sum
DOFs: Degrees of freedom
DRM: Distal resection margin
IIEF-5: 5-Item version of the International Index of Erectile Function
IPSS: International Prostate Symptom Score
LAR: Low anterior resection
LTME: Laparoscopic TME
OT: Operative time
RTME: Robotic TME
TME: Total mesorectal excision
