# Supplementary material for: Comparison of the operative outcomes and learning curves between laparoscopic and “Micro Hand S” robot-assisted total mesorectal excision for rectal cancer: a retrospective study
Source: BMC Gastroenterol. 2021 Jun 7;21:251. doi: 10.1186/s12876-021-01834-1 (PMC8186043; doi:10.1186/s12876-021-01834-1)
Supplement: Supplementary file 1 — Additional file 1. The questionnaires on functional outcomes. [file 12876_2021_1834_MOESM1_ESM.docx]

**
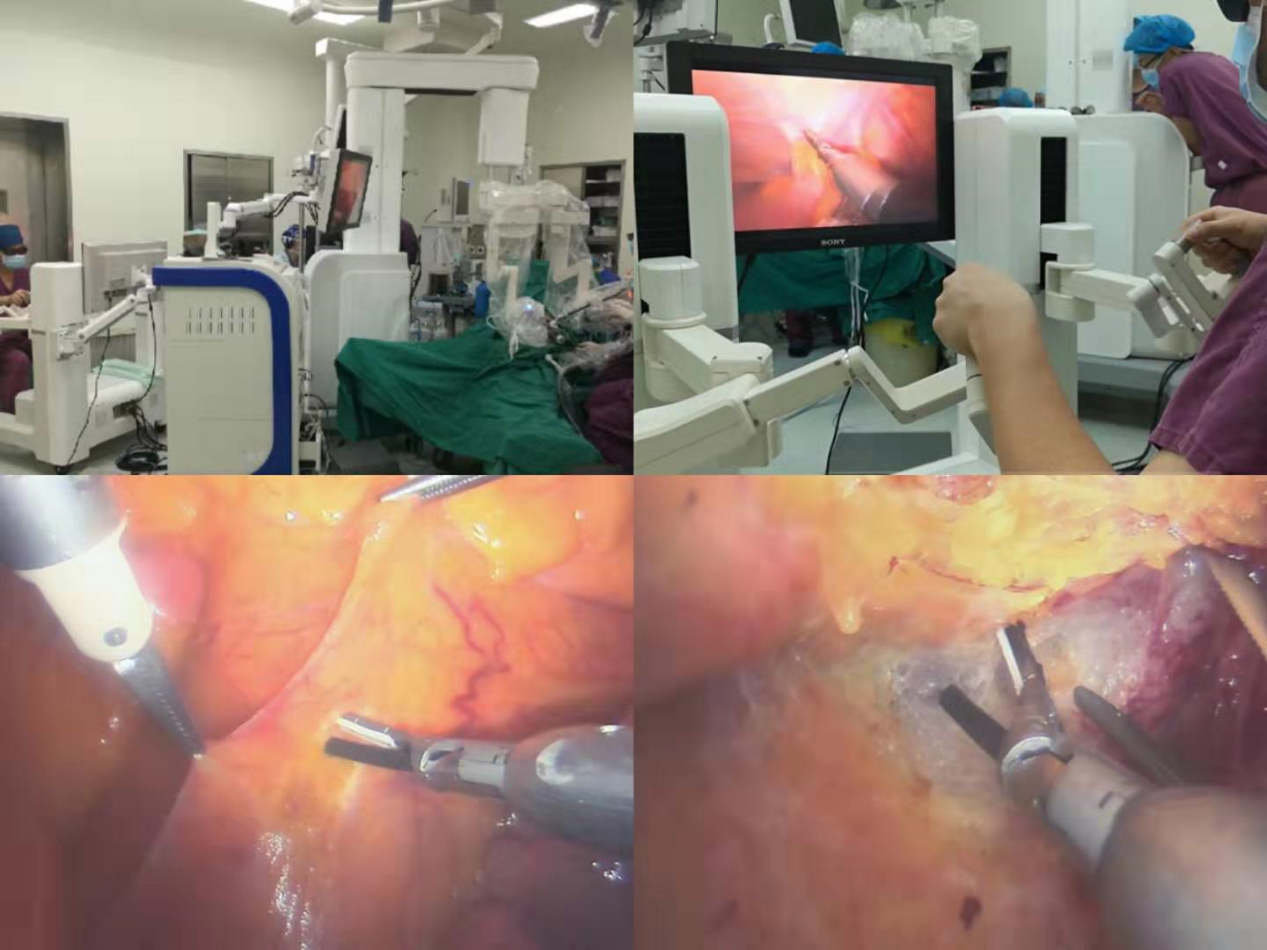
**

**
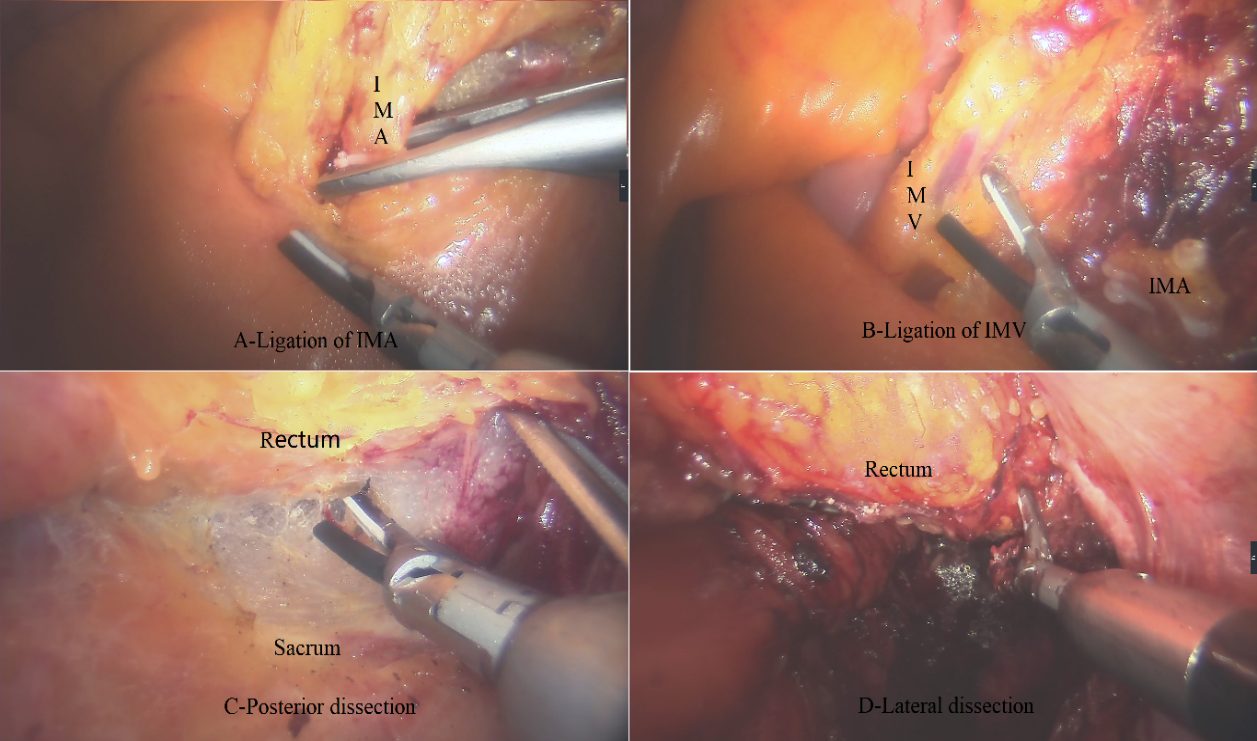
Fig S1.** Pictures of robotic surgery

**Fig S2.** Pictures of surgical process.

**
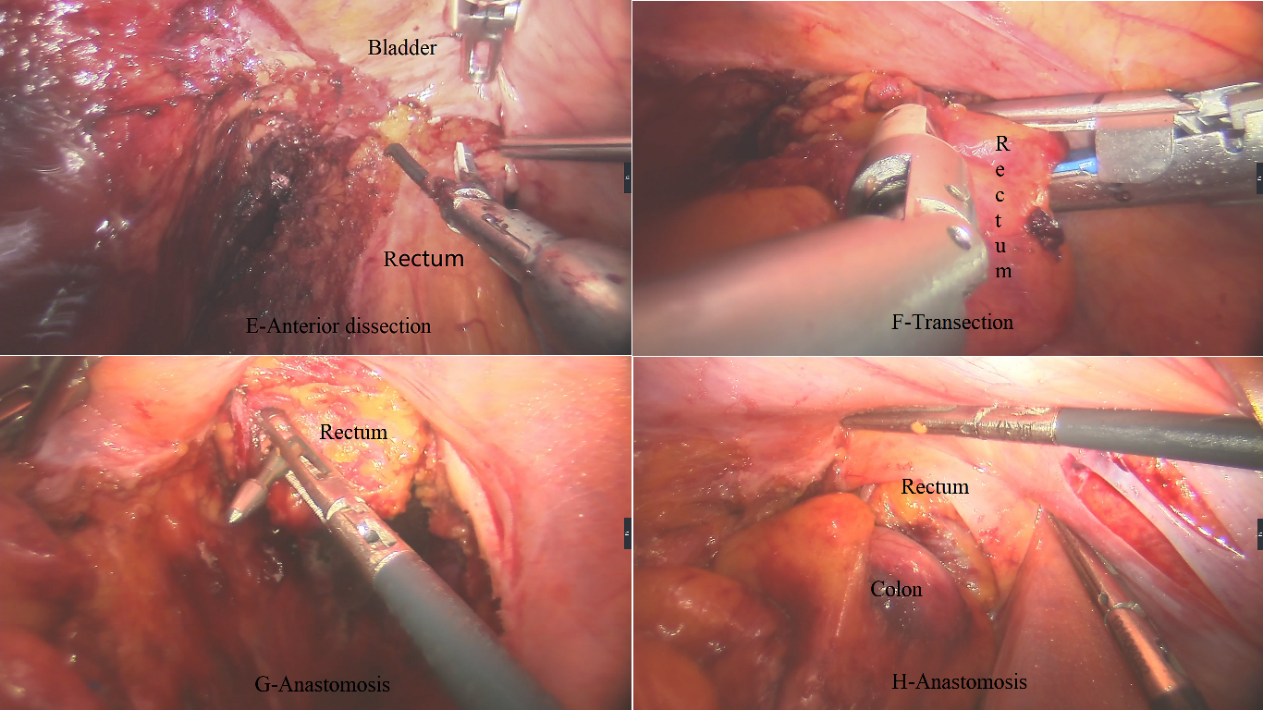
Fig S3.** Pictures of surgical process.

**Video S1**. The movie of manipulation of the surgeon console during operation
