# Supplementary material for: Comparison of the operative outcomes and learning curves between laparoscopic and “Micro Hand S” robot-assisted total mesorectal excision for rectal cancer: a retrospective study
Source: BMC Gastroenterol. 2021 Jun 7;21:251. doi: 10.1186/s12876-021-01834-1 (PMC8186043; doi:10.1186/s12876-021-01834-1)
Supplement: Supplementary file 2 — Additional file 2. The details during the operation. [file 12876_2021_1834_MOESM2_ESM.docx]

**Questionnaire 1** The 5-item version of the International Index of Erectile Function (IIEF-5)

| Item | 1 | 2 | 3 | 4 | 5 | Score |
| --- | --- | --- | --- | --- | --- | --- |
| How do you rate your **confidence** that you could get and keep an erection? | Very low | Low | Moderate | High | Very high |  |
| When you had erections with sexual stimulation, **how often** were your erections hard enough for penetration? | Almost never/never | A few times  (much less than half the time) | Sometimes  (about half the time) | Most times  (much more than half the time) | Almost always/always |  |
| During sexual intercourse, **how often** were you able to maintain your erection after you had penetrated (entered) your partner? | Almost never/never | A few times  (much less than half the time) | Sometimes  (about half the time) | Most times  (much more than half the time) | Almost always/always |  |
| During sexual intercourse, **how difficult** was it to maintain your erection to completion of intercourse? | Extremely difficult | Very difficult | Difficult | Slightly difficult | Not difficult |  |
| When you attempted sexual intercourse, **how often** was it satisfactory for you? | Almost never/never | A few times  (much less than half the time) | Sometimes  (about half the time) | Most times  (much more than half the time) | Almost always/always |  |
| **Total score** | | | | | |  |

The IIEF-5 score is the sum of the ordinal responses to the five items; thus, the score can range from 5 to 25

**Questionnaire 2** The International Prostate Symptom Score (IPSS)

| Question | 0 | 1 | 2 | 3 | 4 | 5 | Score |
| --- | --- | --- | --- | --- | --- | --- | --- |
| During the last month or so, how often have you had a sensation of not emptying your bladder completely after you finished urinating? | Not at all | Less than 1 time in 5 | Less than half the time | About half the time | More than half the time | Almost always |  |
| During the last month or so, how often have you had to urinate again less than 2 hours after you finished urinating? | Not at all | Less than 1 time in 5 | Less than half the time | About half the time | More than half the time | Almost always |  |
| During the last month or so, how often have you found you stopped and started again several ties when you urinated? | Not at all | Less than 1 time in 5 | Less than half the time | About half the time | More than half the time | Almost always |  |
| During the last month or so, how often have you found it difficult to postpone urination? | Not at all | Less than 1 time in 5 | Less than half the time | About half the time | More than half the time | Almost always |  |
| During the last month or so, how often have you had a weak urinary stream? | Not at all | Less than 1 time in 5 | Less than half the time | About half the time | More than half the time | Almost always |  |
| During the last month of so, how often have you had to push or strain to begin urination? | Not at all | Less than 1 time in 5 | Less than half the time | About half the time | More than half the time | Almost always |  |
| During the last month or so, how many times did you  most typically get up to urinate from the time you went to bed at night until the time you got up in the morning? | None | 1 time | 2 times | 3 times | 4 times | 5 or more times |  |
| **Total score** | | | | | | |  |

The total score range from 0 to 35, with a score of 0 to 7 indicating mild symptoms, 8 to 19 indicating moderate symptoms, and 20 to 35 indicating severe symptoms.
